# Remodeling the tumor microenvironment: regulatory effects of β-sitosterol and luteolin on the immunosuppressive milieu in endometrial carcinoma and implications for combinatorial immunotherapy

**DOI:** 10.3389/fimmu.2025.1669606

**Published:** 2025-12-17

**Authors:** Guojie Ji, Pengbo Wang, Zhihong Kong, Xiangxiang Cao, Xiaowei Shi, Huigen Feng, Huanhuan Hu

**Affiliations:** Key Laboratory of Fertility Preservation, North Henan Medical University, Xinxiang, Henan, China

**Keywords:** endometrial carcinoma, tumor immunosuppressive microenvironment, tumor-associated macrophages, phytosterol β-sitosterol, flavonoid luteolin

## Abstract

Endometrial carcinoma (EC), particularly high-risk molecular subtypes like p53abn and NSMP, is frequently characterized by a tumor immunosuppressive tumor microenvironment (TME) that drives progression, metastasis, and resistance to therapy. This immunosuppressive milieu is orchestrated by key cellular components, including M2-polarized tumor-associated macrophages (TAMs), regulatory T cells (Tregs), myeloid-derived suppressor cells (MDSCs), PD-L1-expressing tumor cells, and pro-fibrotic cancer-associated fibroblasts (CAFs), which collectively inhibit effector T cell function and promote immune exclusion/desert phenotypes. Natural products offer promising multi-targeted strategies to remodel the TME. This review comprehensively summarizes the potent immunomodulatory effects of the phytosterol β-sitosterol (BSS) and the flavonoid luteolin (Lut) specifically within the EC TME. We detail how BSS and Lut synergistically reprogram TAMs towards an M1 phenotype, inhibit Treg differentiation/function and MDSC expansion, enhance CD8^+^ T cell recruitment, activation, and cytotoxicity (e.g., by downregulating PD-1/TIM-3), and suppress CAF-mediated immunosuppression and fibrosis. Mechanistically, these effects are achieved through targeting critical signaling pathways (STAT3, NF-κB, PI3K/AKT, Wnt/β-catenin) and modulating key chemokines/cytokines (e.g., reducing TGF-β, IL-10, CXCL12; increasing CXCL9/10, IFN-γ). Critically, BSS and Lut demonstrate significant potential to overcome resistance to immune checkpoint inhibitors (ICIs), particularly in immune-cold EC subtypes. By remodeling the immunosuppressive TME, BSS/Lut combinations can enhance ICI efficacy, as evidenced by preclinical data showing increased tumor suppression rates and T cell infiltration. While challenges remain, including EC-specific validation, bioavailability optimization, and molecular subtype stratification, BSS and Lut represent promising natural adjuvants for combinatorial immunotherapy, offering novel strategies to improve outcomes for patients with aggressive or treatment-refractory EC.

## 1 Overview of EC

EC is one of the most common gynecological malignancies, accounting for 20-30% of all malignant tumors in the female reproductive tract (1). Over recent years, there has been a marked increase in incidence rates, with mortality rates rising even more rapidly (2, 3). EC is traditionally classified into two subtypes: Type I (estrogen-dependent) and Type II (non-estrogen-dependent) (4–6). Type I includes low-grade endometrioid adenocarcinomas (G1/G2), which are linked to obesity and metabolic syndrome (4, 7). This subtype accounts for 70-90% of cases and has a favorable prognosis (5-year overall survival rate >85%). In contrast, Type II includes high-grade endometrioid carcinomas (G3), serous carcinomas, and clear cell carcinomas. These highly aggressive tumors account for only 10% of cases but are responsible for 40% of deaths, with a poor prognosis (5-year survival rate <55%) (2, 4–6, 8, 9). The molecular classification of EC based on The Cancer Genome Atlas (TCGA) further stratifies it into four subtypes (10–13):(1)POLEmut (POLE ultramutated subtype): Characterized by an extremely high mutational burden, these tumors have an excellent prognosis and are classified as low-risk regardless of histopathological grade (12);(2)MSI-H (microsatellite instability-high)/MMRd (mismatch repair deficient subtype): This subtype exhibits an intermediate prognosis and is frequently observed in endometrioid EC (12, 13);(3)NSMP (non-specific molecular profile subtype): Lacking specific molecular signatures, it demonstrates a moderate prognosis (12, 14);(4)p53abn (p53 abnormal subtype): Associated with high-grade/non-endometrioid carcinomas (e.g., serous carcinomas), this subtype carries the poorest prognosis (12, 14, 15). Importantly, this molecular classification has significantly improved prognostic prediction (12).

While TCGA-based molecular classification has improved risk stratification, targeted therapies for the p53abn and NSMP subtypes remain unclear (11, 12, 16). Patients with the p53abn subtype experience high recurrence rates, with metastatic EC in this group having a 5-year survival rate of less than 20% (2, 8, 11). This subtype is also associated with poor responsiveness to conventional chemotherapy and a tendency to develop secondary drug resistance. In contrast, the NSMP subtype also lacks effective targeted therapies (11, 14, 15). Additionally, higher-grade histological types (e.g., serous carcinoma) and lymphovascular space invasion are identified as high-risk factors for recurrence (15, 17). Moreover, therapeutic advancements for advanced or recurrent cases remain limited, particularly in terms of breakthrough therapies for Type II EC (2, 8, 18). Consequently, the primary therapeutic challenges in EC revolve around tumor recurrence, metastasis, and drug resistance. Although international clinical trials like RAINBO are exploring molecular subtype-based individualized adjuvant therapies, further evidence-based support is still needed (12, 13, 16).

## 2 The TME in EC: a “fortress” of immune suppression

The TME is a dynamic ecosystem comprising immune cells (e.g., T cells, dendritic cells, macrophages, tumor-infiltrating lymphocytes [TILs]), stromal cells (e.g., fibroblasts), endothelial cells, inflammatory mediators (e.g., cytokines and chemokines), and extracellular matrix molecules (19–21). These components form an interactive network modulating the balance between immune suppression and activation, influencing tumor progression and therapeutic responses. The composition, spatial distribution, and functional status of the TME provide both theoretical foundations and therapeutic targets for precision immunotherapy (19, 20, 22, 23). EC is classified into four immune phenotypes based on immune cell infiltration: C1 (immune-suppressed), C2 (IFN-γ-dominant), C3 (inflammatory, exhibiting the most robust immune responses), and C4 (lymphocyte-depleted) (19, 24). These immune phenotypes differentially impact therapeutic strategies and prognosis: the C3 subgroup exhibits the lowest risk scores and favorable immune activity (e.g., T cell activation), whereas the C4 subgroup shows the highest risk and poorest immune activity (24).

**Figure 1:**
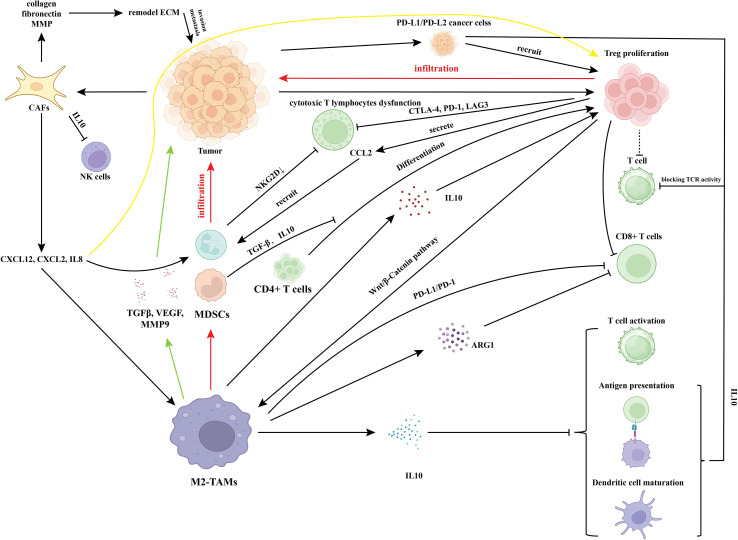
The TME in EC.

Tumor cells evade immune surveillance by orchestrating the TME. Studies demonstrate that tumor cells promote Tregs expansion to suppress CD8^+^ T cell function (23, 25, 26). Metabolic reprogramming critically regulates immune responses: glycolytic lactate production acidifies the microenvironment, suppressing immune cell activity and facilitating immune evasion (22, 26). Moreover, immune cell spatial distribution (e.g., IDO1^+^ macrophage-CD8^+^ T cell co-localization) dictates immune response magnitude (27, 28). Elevated TTK (tumor tissue kinase) expression correlates with reduced immune scores and decreased infiltration of multiple immune cell subsets, contributing to an immunosuppressive TME (29). TTK can alter the composition of tumor-infiltrating immune responses. Studies have shown that high TTK expression is positively correlated with increased infiltration of Th2 cells, while it is negatively correlated with infiltration of CD56 bright NK cells. This change in immune cell composition contributes to the formation of an immunosuppressive microenvironment. Furthermore, the enrichment scores of multiple types of infiltrating immune cells are negatively correlated with elevated TTK expression. This indicates that high TTK expression is accompanied by a widespread reduction in immune cells, which reduces overall immune activity and further impairs the anti-tumor immune response in the TME (29). Tumor cells evade immune surveillance through specific signaling cascades (e.g., MDL-NCL pathway) and microenvironmental remodeling, impairing immune cell function (21). The MDK-NCL signaling pathway refers to a ligand-receptor pair composed of Midkine (MDK) as the ligand and Nucleolin (NCL) as the receptor, which exerts its function in tumors such as EC by activating the MK signaling pathway (21, 30). MDK is mainly expressed in endometrial carcinoma cells and ciliated cells, while NCL is widely expressed in various cell types. They form a key ligand-receptor pair that transmits signals in intercellular communication, enabling epithelial cells to transfer malignant phenotypes to endothelial cells, helping them escape immune surveillance and promoting tumor progression. Enrichment of quiescent CD4^+^ T cells and activated dendritic cells correlates with favorable prognosis. Conversely, high M2-type macrophage infiltration correlates with poor prognosis in high-risk subgroups (28, 29). High CD8^+^ T cell infiltration sensitizes tumors to immunotherapy (19), while absent infiltration impairs immune responses (19, 29). Collectively, these findings demonstrate that tumor immune heterogeneity directly impacts clinical outcomes (**Figure 1**).

### 2.1 TAMs: M2 macrophage polarization dominates the secretion of immunosuppressive factors (IL-10, TGF-β, ARG1, iNOS), suppressing T cell function and promoting angiogenesis/metastasis

Within the TME, TAMs predominantly exhibit an immunosuppressive M2 phenotype. This polarization is driven by tumor cell-secreted factors and chemokines, reinforced through STAT3 and NF-κB signaling pathways, establishing an immunosuppressive milieu that promotes tumor growth (31–34). M2-polarized TAMs highly express immunosuppressive factors including interleukin-10 (IL-10), TGF-β, arginase 1 (ARG1), and inducible nitric oxide synthase (iNOS). Specifically, IL-10 inhibits dendritic cell maturation and antigen presentation, impairing T cell activation (35, 36). TGF-β directly induces T cell dysfunction, promotes regulatory T cell (Treg) expansion, and reinforces M2 polarization through the Wnt/β-catenin pathway (31, 37). ARG1 and iNOS deplete microenvironmental arginine, suppressing CD8^+^ T cell metabolic activity and cytotoxic function (38–40). TAMs directly suppress CD8^+^ T cell activation and cytotoxicity through checkpoint molecules (e.g., PD-L1/PD-1) or secretion of IL-10 and TGF-β (38–41). Simultaneously, they activate immunosuppressive cells, facilitating MDSC and Treg infiltration, thereby establishing an immune-excluded microenvironment (41, 42). Additionally, TAMs secrete pro-angiogenic factors including VEGF, MMP-9, and TGF-β to induce endothelial cell proliferation and angiogenesis. They enhance tumor nutrient supply and invasiveness through extracellular matrix (ECM) remodeling and epithelial-mesenchymal transition (EMT) pathway activation (33, 35, 37). Furthermore, TAMs mediate chemoresistance (e.g., to cisplatin) and interact with cancer stem cells to sustain metastatic potential (31, 33, 40).

### 2.2 Tregs: suppression of effector T cell (Teffs, CTLs) activation and function

Tregs, characterized by CD25^+^ expression and the key transcription factor FoxP3^+^, are a CD4^+^ T lymphocyte subset with immunosuppressive functions (42).Under physiological conditions, Tregs maintain immune homeostasis by suppressing effector T cell activity; however, their TME expansion promotes immune evasion (42, 43). Tregs inhibit cytotoxic T lymphocytes (CTLs) through direct and indirect mechanisms. Through direct interactions, Tregs suppress CTL activation via cell-to-cell contact. Tregs highly express immune checkpoint molecules (e.g., CTLA-4, PD-1, LAG-3) that block CTLs co-stimulatory signals and impair cytotoxic function (9, 44). Through indirect mechanisms, Tregs secrete immunosuppressive factors inducing CTLs anergy and promoting an exhausted phenotype (9, 37, 39). Tregs orchestrate the TME by depleting local IL-2 to limit CTLs survival/proliferation while relying on IL-2 to sustain their immunosuppressive functions, creating a negative feedback loop (9, 39). Tregs synergize with MDSCs to reinforce local immunosuppression, contributing to T-cell exclusion (38, 39, 41). Tregs drive T cell exhaustion and dysfunction to inhibit CTLs. They induce effector T cell exhaustion (e.g., PD-1^high^ HAVCR2^high^ CD8^+^ T_EX_ cells) while suppressing cytotoxic T cell (CD8^+^ T_CYTO_) differentiation (9, 44). Tregs activate immunosuppressive pathways (e.g., NF-κB) via transcription factors (FOXP3, RUNX1), further dampening CTL effector functions and exacerbating T cell exhaustion (9, 40). Increased Treg infiltration correlates with poor prognosis in solid tumors including EC. Tregs promote tumor progression by suppressing effector T cells and reshaping the immunosuppressive microenvironment, thereby driving immune checkpoint inhibitor (ICI) resistance.

### 2.3 MDSCs: inhibition of T/NK cells and promotion of Tregs

MDSCs are a heterogeneous population of bone marrow-derived myeloid cells with potent immunosuppressive functions. These cells exist in an immature state and expand or accumulate in peripheral blood and the TME under pathological conditions (42). MDSCs induce apoptosis or functional inactivation of T cells and natural killer (NK) cells by depleting essential nutrients through secretion of arginase-1 (Arg-1), nitric oxide (NO), and reactive oxygen species (ROS) (37, 42). Specifically, MDSCs block T cell cycle progression at the G0/G1 phase and inhibit T cell receptor (TCR) signaling through arginine degradation via Arg-1 (37, 42). Concurrently, they suppress NK cell cytotoxicity and interferon-gamma (IFN-γ) production by downregulating activation receptors (e.g., NKG2D) (28, 42). Similar to TAMs, MDSCs induce naive CD4^+^ T cell differentiation into FOXP3^+^ Tregs via TGF-β and IL-10 secretion (42). Tregs reciprocally secrete chemokines including CCL2 to recruit MDSCs into the TME, while MDSCs expand the Treg population, collectively establishing an immune-excluded microenvironment (42). This reciprocal interaction establishes a positive feedback loop between MDSCs and Tregs. In microsatellite-stable (MSS) tumors, synchronous increases in MDSCs and effector Tregs (eTregs) contribute to immunotherapy resistance (42). Collectively, MDSCs promote immune tolerance through T cell suppression and play a pivotal role in mediating TME immunosuppression.

### 2.4 Immunosuppressive cancer cells: expression of PD-L1/PD-L2

Cancer cells evade immune surveillance by suppressing immune cell function. Tumor cells highly express immune checkpoint ligands like PD-L1, which bind to PD-1 receptors on T cells, transmitting inhibitory signals that induce T cell exhaustion or anergy (23, 45–47). PD-L1 expression is regulated by both endogenous oncogenic signals (e.g., EGFR, IRF1) and exogenous immune stimuli (e.g., IFN-γ) (48). PD-L2, another PD-1 ligand, shares expression patterns with PD-L1 but has restricted distribution, being primarily enriched in dendritic cells and less expressed in tumor cells (42, 49). In EC, PD-L1 expression is significantly elevated (25–40%) in mismatch repair-deficient (dMMR) or MSI-H subtypes, but lower in microsatellite-stable (MSS) tumors (23). In POLE-mutant tumors, PD-L1 expression positively correlates with T cell infiltration, contributing to an immune-inflamed microenvironment (23).

The primary immunosuppressive mechanism involves PD-L1/PD-L2 binding to PD-1 on T cells, which (1): blocks TCR activation and co-stimulatory pathways (e.g., CD28/B7) (2); induces T cell anergy and exhaustion (characterized by LAG-3/TIM-3 overexpression); and (3) inhibits cytotoxic factor release (23, 42). and inhibits the release of cytotoxic factors (23, 50). This leads to an immune-excluded microenvironment where T cells are present but functionally inert (23). PD-L1^+^ tumor cells recruit Tregs, enhancing their immunosuppressive functions (42, 48), and suppress dendritic cell maturation/antigen presentation via soluble factors (e.g., IL-10), indirectly weakening antitumor immunity (49, 51).

High tumor cell PD-L1 expression correlates with advanced lymph node metastasis and poor disease-free survival (DFS), especially in MSS subtypes (23). Conversely, PD-L1 expression on immune cells correlates with increased lymphocyte infiltration, potentially predicting better immunotherapy response (48, 50, 52). Different scoring systems—Combined Positive Score (CPS), Tumor Proportion Score (TPS), and Immune Cell Score (IC)—differ in predictive value. CPS integrates PD-L1 expression on tumor and immune cells, demonstrating superior predictive efficacy for PD-1 inhibitor responses (23).

### 2.5 CAFs: shaping a pro-fibrotic and immunosuppressive stroma, and secreting chemokines such as CXCL12 to recruit immunosuppressive cells

CAFs are activated, heterogeneous fibroblasts within the TME, primarily derived from normal tissue fibroblasts transformed by tumor-derived signals (e.g., cytokines and growth factors). As the dominant stromal component in the TME (9, 35, 37, 53), CAFs express characteristic surface markers: α-smooth muscle actin (α-SMA), fibroblast activation protein (FAP), and CD146. The CD146^+^ CAF subpopulation is significantly associated with poor prognosis (9, 35).CAFs remodel the extracellular matrix (ECM) through collagen, fibronectin, and matrix metalloproteinase (MMP) secretion, increasing tissue stiffness to promote tumor invasion and metastasis (9, 35, 53). Additionally, IL-10/JAK1/STAT3 pathway activation induces vasculogenic mimicry, forming vasculogenic mimicry that support tumor blood supply (35). Vasculogenic mimicry—a tubular structure capable of transporting blood, formed by invasive tumor cells themselves through deformation and extracellular matrix remodeling. The inner lining of these tubules is composed of tumor cells rather than endothelial cells, and the tumor cells covering the tubules express certain endothelial cell markers (e.g., VE-cadherin), a process similar to EMT. Vasculogenic mimicry is a characteristic of invasive tumors such as melanoma and is closely associated with tumor metastasis and poor prognosis (35, 54). Myofibroblast-like CAFs (myoCAFs) expressing matrix metalloproteinase-11 (MMP11) and tenascin C (TNC) drive matrix sclerosis and tumor dissemination (9, 53).

CAFs shape immunosuppressive microenvironments by secreting chemokines that recruit immune cells and directly inhibiting effector immune functions. CAFs recruit MDSCs, Tregs, and M2 macrophages via chemokine secretion (e.g., CXCL12/SDF-1, CXCL2, IL-8) (26, 55). CXCL12 enhances MDSC immunosuppressive activity through CXCR4 receptor binding (55). Furthermore, CAF-secreted IL-10, reactive oxygen species (ROS), and arginase-1 (Arg-1) directly inhibit CD8^+^ T cell proliferation and function, promoting T cell exhaustion (34, 55). CAF-derived interleukin-6 (IL-6) inhibits natural killer (NK) cell cytotoxicity and interferon-gamma (IFN-γ) production, impairing antitumor immunity (37, 56).

Although most CAFs (e.g., CD146^+^ subtypes) promote tumor progression (35), certain subtypes (e.g., Hedgehog-activated CAFs) inhibit matrix sclerosis to delay tumor growth (55). Targeting CAF secretory pathways (e.g., IL-10/JAK1/STAT3 or CXCL12/CXCR4 blockade) reverses immunosuppression (35, 55), while FAP inhibition reduces Treg infiltration and enhances chemotherapy sensitivity (37, 57).

### 2.6 The distribution patterns of TAMs, Tregs, and MDSCs across EC subtypes

Multiple studies have shown that the frequency of monocytic myeloid-derived suppressor cells (M-MDSCs) in the peripheral blood and tumor tissues of patients with MSS EC is significantly higher than that in patients with MSI-high EC, with also higher M-MDSC infiltration. Regardless of the treatment received, the increase in activated/effector Tregs in MSS-type EC patients is associated with the MSS status. Evidence indicates that p53abn EC usually has fewer TILs and belongs to the “immune desert” type of tumors, which also suggests an immunosuppressive microenvironment (42). In summary, existing evidence indicates that MDSCs (M-MDSCs) and Tregs are mainly enhanced in MSS-type EC. Although the p53abn subtype often exhibits an immunosuppressive microenvironment, consistent with the immunosuppressive function of TAMs, there is no evidence that TAMs, Tregs, or MDSCs are specifically and predominantly enhanced in type II or p53abn EC.

## 3 Immune-excluded/desert phenotypes: T cell infiltration deficiency in some EC

Immune phenotypes are classified into three principal categories: immune-excluded, immune-desert, and immune-inflamed. The immune-desert phenotype features near-absent T cell infiltration (especially CD8^+^ T cells) in tumor tissues, with stroma dominated by pro-tumor components including blood vessels, fibroblasts, and macrophages (9, 41). The immune-excluded phenotype is defined by peripheral T cell accumulation without core infiltration (41). Conversely, the immune-inflamed phenotype shows abundant CD8^+^ TILs within tumor parenchyma, indicating active antitumor immunity (19, 58, 59). Approximately 60% of non-specific molecular profile (NSMP) subtypes exhibit the immune-desert phenotype with minimal T cell infiltration (19, 58). The p53abn subtype typically presents an immune-excluded phenotype with T cells confined to margins but absent from the core (58). POLE-mutant and dMMR/MSI-H subtypes show significant enrichment in the immune-inflamed phenotype, exhibiting the highest CD8^+^ TIL densities (58–60).

Multiple mechanisms drive immune-desert phenotype formation (1): high GRHL1 expression suppressing CD8^+^ T cell recruitment/activation (41); (2) MYC target gene dysregulation and type I IFN pathway inactivation impairing chemokine secretion, reducing T cell infiltration (22, 58); (3) major histocompatibility complex class I (MHC-I) downregulation causing defective antigen presentation and T cell evasion (9, 40, 47) (4). CAFs secreting TGF-β and C-X-C motif chemokine ligand 12 (CXCL12) to form fibrotic barriers blocking T cell migration (44, 61, 62) (5); metabolic disorders in hypoxic tissues and lactate accumulation causing acidification that suppresses T cell function and promotes exhaustion (9, 60, 63) (6); in MSS tumors, DNA hypermethylation silencing C-X-C motif chemokine ligand 9 (CXCL9) and CXCL10 expression, blocking T cell recruitment (19, 59, 64).

ICIs block checkpoint molecules (e.g., programmed death-1/programmed death-ligand 1 [PD-1/PD-L1] axis, cytotoxic T-lymphocyte-associated protein 4 [CTLA-4]) to restore T cell antitumor activity. By disrupting tumor-derived inhibitory signals, ICIs restore immune recognition and cancer cell elimination. ICI responses vary by phenotype: the immune-inflamed phenotype (high CD8^+^ TILs) shows significantly higher objective response rates (ORR >40%), particularly in dMMR/MSI-H subtypes (58, 65). Conversely, immune-desert phenotypes (peripherally sequestered or absent T cells) show low response rates to single-agent ICIs (<15%) (58, 65). In immune-desert phenotypes, stromal cells (CAFs, Tregs) comprise >50% of tissue; their TGF-β secretion inhibits CD8^+^ T cell cytotoxicity, reducing anti-PD-1 efficacy (23, 65, 66).

Combination therapies can remodel the TME to enhance ICI efficacy: Stereotactic body radiation therapy releases tumor antigens, activates dendritic cells, and improves T cell infiltration to reverse physical barriers (65). Vascular endothelial growth factor (VEGF) inhibitors normalize vasculature to overcome T cell trafficking barriers (67–69). Low-dose cyclophosphamide depletes infiltrating Tregs, enhancing antigen presentation (65); DNA methyltransferase (DNMT) inhibitors restore CXCL9/CXCL10 expression, promoting effector T cell recruitment (26, 68); Neutralizing agents (e.g., sodium bicarbonate) reverse lactate-induced acidification, restoring T cell cytotoxicity (65). Thus, the TME critically regulates ICI efficacy, offering therapeutic opportunities through CAF/Treg targeting and barrier dismantling to improve outcomes.

## 4 Natural products: a new hope for remodeling the immune microenvironment

Natural products reverse immunosuppression through multi-targeted mechanisms. Flavonoid-based natural products target immunosuppressive cells by: downregulating HIF-1α/STAT3 signaling in TAMs; blocking M2 polarization (odds ratio [OR]=3.2); and reducing IL-10 secretion (33, 70). Traditional Chinese medicine (TCM) formulas deplete Tregs decrease TGF-β levels, and restore CD8^+^ T cell cytotoxicity (increasing clinical response rates by 40%) (34, 71). C Certain natural products disrupt metabolic immunosuppression by antagonizing CAF-secreted lactate, reversing acidification (pH 6.5→7.2), and alleviating T cell dysfunction (34, 60).

**Figure 2:**
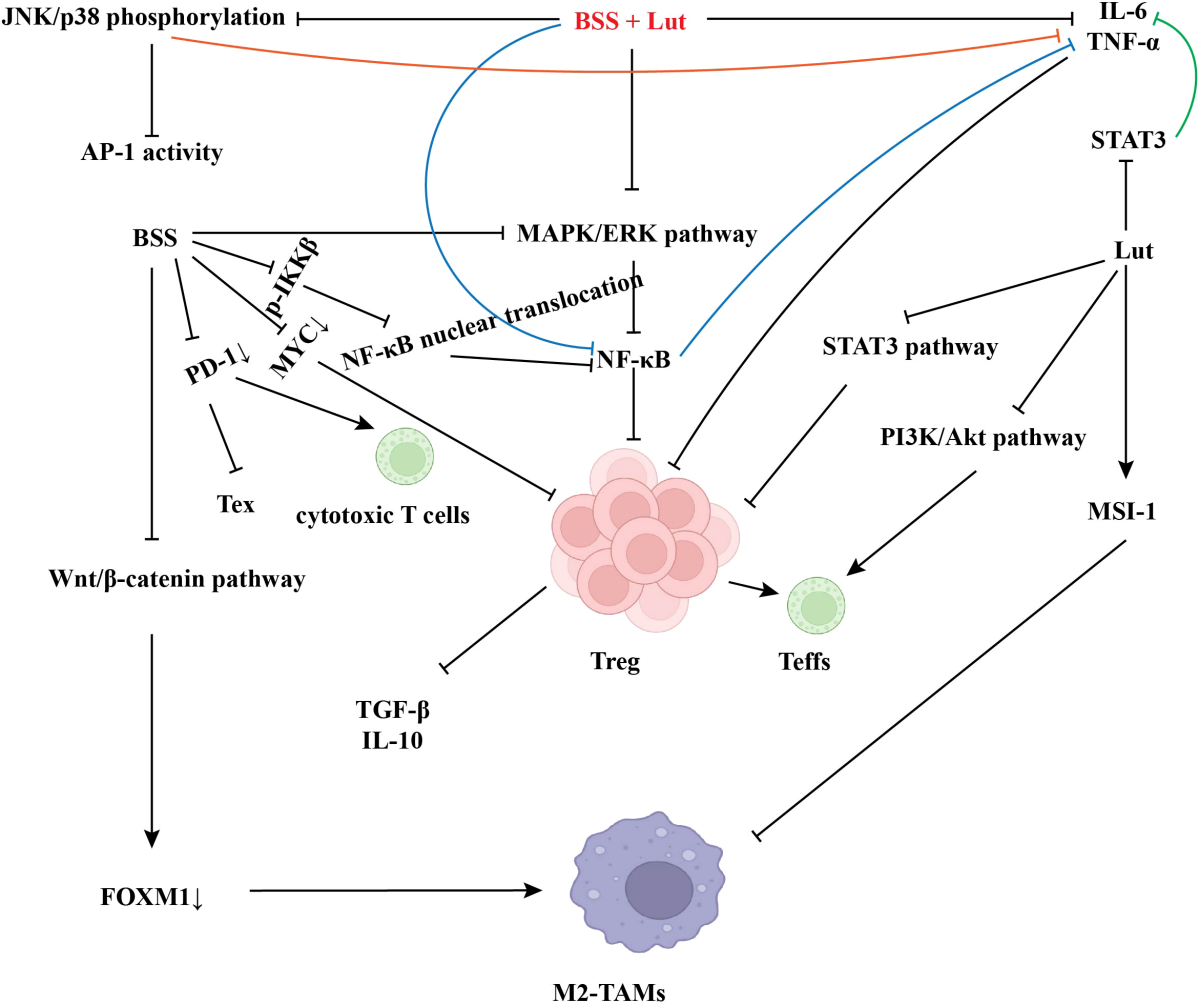
Natural products remodel the immune microenvironment.

The flavonoid kaempferol reverses doxorubicin resistance in breast cancer cells by inhibiting nuclear factor kappa B (NF-κB) signaling (60% reduction in half-maximal inhibitory concentration [IC_50_]) (34, 72, 73). TCM formulas downregulate programmed death-ligand 1 (PD-L1) via PI3K/AKT signaling, enhancing ICIs response rates (objective response rate [ORR] 26%→52%) (74, 75). Flavonoids increase tumor radiosensitivity by promoting reactive oxygen species (ROS)-mediated DNA damage (70, 76). However, natural products exhibit extremely low *in vivo* bioavailability; modified delivery systems enhance absorption and therapeutic efficacy. Liposomal delivery of Lut increases bioavailability from 8% to 75% (77, 78). TCM modernization faces bottlenecks from natural product complexity. Multi-component TCM mechanisms require network pharmacology analysis (71, 74, 75) and correlation systems linking ingredient fingerprints to TME biomarkers (74, 75). Collectively, natural products remodel immunosuppressive TME ecology, sensitize conventional therapies, and synergize with ICIs (**Figure 2**). Future research should integrate nanotechnology and multi-omics to advance phytochemical applications in precision immunotherapy (34, 74, 75, 77).

### 4.1 Comparative analysis of BSS/Lut with other natural adjuvants in EC

As a representative phytosterol, current studies on BSS have focused on its anti-inflammatory and immunosuppressive properties. Research has shown that BSS exerts anti-inflammatory effects by inhibiting the NF-κB, p38 MAPK signaling pathway, RIG-I signaling pathway, and NLRP3 inflammasome (79). A computational model predicted its potential as a citrus-derived adjuvant to enhance immune system responses, which was initially confirmed in *in vivo* experiments (80). Another study demonstrated that it can stimulate the proliferation of human peripheral blood lymphocytes, suggesting immunomodulatory potential (81). However, all animal studies have used inflammatory or tumor models, and no quantitative evaluation of antigen-specific antibody titers or T cell responses when BSS is used as a vaccine adjuvant has been reported.

In contrast, Lut, when used as an adjuvant, effectively enhances the anti-tumor response of CTLs in the B16F10 mouse melanoma model (82). This study indicated that it can promote the shift of the Th1/Th2 balance toward Th1 and enhance cellular immunity. Its molecular mechanism is similar to that of quercetin: luteolin can inhibit NF-κB activation (83), a property that may be contradictory in the context of vaccines—NF-κB is a key transcription factor for dendritic cell (DC) maturation, and its inhibition may weaken the adjuvant effect.

Quercetin exhibits contradictory immunostimulatory and immunosuppressive mechanisms. Studies have shown that in ovalbumin-immunized mouse models, quercetin displays adjuvant activity by enhancing Th2-type immune responses (84). Its ability to stimulate GM-CSF secretion could theoretically promote DC survival and differentiation (85). However, multiple studies have clearly demonstrated that quercetin inhibits DC activation and function, as well as LPS-induced inflammatory responses (86). It significantly inhibits the production of proinflammatory cytokines such as TNF-α, IL-1β, and IL-6 by regulating the SIRT1/p65 axis (87) and exerts anti-inflammatory effects in ulcerative colitis models (88).

Among the four compounds, kaempferol has the clearest clues regarding its adjuvant mechanism, but lacks systematic evaluation. *In vivo* studies have shown that it possesses adjuvant activity, as it can enhance the expression of transcription factors such as Tbx-21 and GATA-3, and promote DC infiltration (89). This is highly consistent with the DC recruitment and activation required for vaccine adjuvants. Interestingly, kaempferol can also stimulate GM-CSF secretion, but its effects on Th1-polarizing factors such as IL-12 and IFN-γ have not been reported. Meanwhile, there are contradictory indications that it may impair DC immune function (90).

### 4.2 BSS and Lut: multifaceted immunomodulators

BSS is a phytosterol—a natural lipid with a steroidal nucleus featuring a cyclopentanoperhydrophenanthrene skeleton and lipophilic side chain (C_2950_O) ( (–)-beta-Sitosterol | C29H50O | CID 222284 - PubChem) (91–93). BSS is primarily derived from hawthorn species (e.g., Crataegus orientalis, C. pinnatifida) (93) and found in foods (soybean oil, grains, nuts, berries) and Chinese herbal medicines (Astragalus membranaceus, Angelica sinensis) (91, 92, 94). Lut, a flavonoid with molecular formula C_15_H_10_O_6_ (MW: 286.24 g/mol) (Luteolin | C15H10O6 | CID 5280445 - PubChem) (95), exhibits high polarity, ethanol solubility, poor aqueous solubility, and antioxidant polyphenolic hydroxyl groups (33, 95). Lut occurs naturally in vegetables/fruits (e.g., celery, carrots, citrus) and medicinal plants (Schizonepeta tenuifolia, Ajuga decumbens) (94, 95), and is active in TCM formulas like Guben Zenggu Granules (94).

BSS and Lut inhibit M2 polarization as a primary therapeutic mechanism. BSS suppresses M2 polarization by inhibiting Wnt/β-catenin signaling, reducing forkhead box M1 (FOXM1)-mediated M2 marker expression (96). In glioblastoma, Lut binds Musashi-1 (MSI-1), inhibiting macrophage M2 polarization and attenuating pro-tumor functions (97). BSS and Lut synergistically enhance immune regulation by modulating ATP-binding cassette (ABC) transporters and activating p53-dependent pathways.

BSS and Lut suppress regulatory T cell (Treg) differentiation/function while activating effector T cells (Teffs). BSS inhibits NF-κB activation via mitogen-activated protein kinase/extracellular signal-regulated kinase (MAPK/ERK) downregulation, reducing TGF-β/IL-10 secretion and Treg proportions in the TME (94, 98). Lut inhibits signal transducer and activator of transcription 3 (STAT3)—critical for Treg function—blocking immunosuppression (33), and reducing inhibitory receptors (e.g., cytotoxic T-lymphocyte-associated protein 4 [CTLA-4]) on Tregs, relieving Teff suppression (33, 42). BSS reverses T cell exhaustion by downregulating programmed death-1 (PD-1) expression (30% reduction), enhancing CD8^+^ T cell cytotoxicity (91, 92); Lut promotes interferon-gamma (IFN-γ) secretion, strengthening Teff antitumor activity (65, 74). Lut blocks hypoxia-inducible factor-1α (HIF-1α) signaling, improving TME hypoxia and restoring Teff metabolic function (33, 71).

BSS and Lut synergistically regulate immune balance. In Guben Zenggu Granules, they synergistically inhibit Treg expansion by downregulating interleukin-6/tumor necrosis factor-alpha (IL-6/TNF-α) while activating Teff pathways (94). BSS inhibits MYC signaling to impair Treg survival, while Lut blocks phosphatidylinositol 3-kinase/protein kinase B (PI3K/AKT) to promote Teff proliferation, forming a dual mechanism (54, 74). Collectively, BSS and Lut remodel T cell balance by targeting NF-κB/STAT3 to inhibit Treg differentiation, activate Teffs, enhance IFN-γ, and reverse exhaustion. Clinical applications require targeted delivery and formula optimization for improved specificity (33, 91, 98).

BSS and Lut target NF-κB and MAPK pathways to inhibit TNF-α and IL-6 secretion. BSS blocks NF-κB nuclear translocation by inhibiting inhibitor of NF-κB kinase subunit beta (IKKβ) phosphorylation, reducing TNF-α (94, 99). Lut binds the Src homology 2 (SH2) domain of STAT3, inhibiting activation and IL-6 transcription (33, 74). They synergistically inhibit JNK/p38 phosphorylation, impairing activator protein-1 (AP-1) activity and suppressing TNF-α (74, 95). BSS and Lut exhibit tissue-specific effects: BSS preferentially inhibits bone metabolism-related inflammation (94), while Lut shows greater TNF-α suppression in the TME (33, 54). BSS and Lut inhibit TNF-α/IL-6 via distinct mechanisms (BSS: NF-κB; Lut: STAT3) with synergistic MAPK blockade.

Studies have indicated that β-sitosterol exerts a significant inhibitory effect on human lung adenocarcinoma cells without damaging normal lung tissue cells (100), suggesting potential selective toxicity towards tumor cells versus normal cells. Additionally, β-sitosterol can inhibit the growth and metastasis of hepatocellular carcinoma by regulating the FOXM1/Wnt/β-catenin pathway (96). Its effects are more often described as acting directly on tumor cells, exerting antitumor effects through mechanisms such as inducing cell cycle arrest, apoptosis, and inhibiting metastasis (92, 93, 100).

Luteolin has been reported to exert antitumor immune effects by promoting the infiltration of CD8+ T lymphocytes (77). In the B16F10 mouse model, luteolin, as an adjuvant, can effectively enhance the antitumor response of CTLs (77). Luteolin is believed to combat tumors by regulating immune cell functions and preventing tumor cell immune escape (70). Despite luteolin’s demonstrated regulatory effects on tumor immunity, there is no evidence that it “selectively” regulates immune cells in tumor tissues versus normal tissues.

### 4.3 Impact of BSS and Lut on key immune signaling pathways

BSS inhibits tumor cell proliferation and immune evasion by blocking β-catenin nuclear translocation, reducing downstream target gene expression (96). BSS indirectly inhibits PI3K signaling, inducing lung cancer cell apoptosis (100). Lut reduces PI3K/AKT phosphorylation, inhibiting prostate cancer cell proliferation and migration (59). Combined with quercetin, Lut enhances AKT inhibition to block glycolytic reprogramming (59). Lut binds the SH2 domain of signal transducer and activator of STAT3, suppressing activation, blocking immunosuppressive cytokine secretion, and downregulating M2 polarization (59, 101). This enhances anti-tumor T cell activity, remodeling the immune microenvironment (40, 101). Lut reverses EMT by inhibiting NF-κB/zinc finger E-box binding homeobox 1 (ZEB1), reducing metastasis in triple-negative breast cancer (59, 102).

BSS and Lut synergistically regulate the immune microenvironment. They downregulate PD-L1 expression by inhibiting MYC signaling, reversing T cell exhaustion, enhancing CD8^+^ T cell infiltration in esophageal squamous cell carcinoma, and blocking immune evasion (59). In traditional Chinese medicine (TCM) formulas (e.g., Guben Zenggu Granules), BSS and Lut synergistically target IL-6/TNF-α/STAT3 to suppress inflammatory microenvironments (96, 101). Collectively, BSS and Lut regulate tumor proliferation, immune evasion, and inflammatory microenvironments by targeting Wnt/β-catenin, MAPK, PI3K/AKT, and STAT3 pathways. Their synergy enhances anti-tumor immune responses (59, 99).

The PI3K/AKT signaling pathway plays a crucial role in the pathogenesis of EC. Among solid tumors, endometrial carcinoma has the highest rate of alterations in the PI3K/AKT/mTOR pathway, with specific alterations in this pathway observed in 92% of type I and 60% of type II endometrial carcinomas (75). Loss of PTEN function leads to overactivation of the PI3K/AKT pathway, thereby stimulating cell proliferation and tumorigenesis (103). This pathway regulates cancer cell apoptosis by influencing downstream activities and is involved in malignant biological behaviors such as tumor proliferation and apoptosis (75). In type II EC, the PI3K/AKT/mTOR signaling pathway is significantly activated, and its enhanced signaling is associated with disease progression and poor prognosis in patients (9).

In EC, the Wnt/β-catenin signaling pathway is the second most frequently activated pathway. Abnormal activation of this pathway is associated with numerous growth-related pathologies and cancer types. β-catenin is a key downstream effector of this pathway, and mutations in its encoding gene CTNNB1 have been observed in endometrial hyperplasia. Translocation of β-catenin from the membrane to the nucleus results in activation of Wnt/β-catenin signaling (104). Studies have confirmed that endometrium-derived mesenchymal stem cells (eMSCs) inhibit EC cell proliferation and stemness through DKK1-Wnt/β-catenin signaling (105). Additionally, changes in β-catenin or components of the degradation complex have been verified as early events in EC (106).

The NF-κB pathway exerts important functions in tumor progression. Evidence indicates the existence of a non-classical estrogen signaling pathway in EC, where NF-κB serves as a key factor (107). Overactivation of NF-κB can be induced by various stimuli, including those through the MAPK/ERK pathway (34). In EC, NF-κB signaling plays a critical role in cancer stem cell phenotypes by regulating key target genes (10) (such as inhibitors of apoptosis proteins, cytokines, and EMT transcription factors). NF-κB activity is also associated with other key signaling pathways such as JAK/STAT3 (34).

Activation of the STAT3 signaling pathway is associated with poor treatment outcomes and increased resistance to chemotherapy and radiotherapy (108). In EC, leptin exerts its effects mainly through the JAK/STAT signaling pathway, which regulates the expression of ERK1/2 signaling, anti-apoptotic proteins (e.g., XIAP), inflammatory proteins (TNF-α, IL-6), angiogenic factors, and hypoxia-inducible factor-1α (49). The impact of persistently activated JAK/STAT signaling on carcinogenesis makes this pathway an important target for new drug development and effective personalized management of breast cancer. Activation of the NF-κB and STAT3 pathways is associated with proliferation and anti-apoptosis of triple-negative breast cancer cells (34).

These pathways are interconnected and collectively promote the occurrence, development, metastasis, and therapeutic resistance of EC. For example, NF-κB exhibits crosstalk with signaling pathways such as STAT3 and PI3K/AKT (34); the PI3K/AKT pathway is also closely associated with pathways like MAPK (75).

## 5 BSS and Lut: direct effects on core immune components in the EC TME

### 5.1 Targeting TAMs

**Figure 3:**
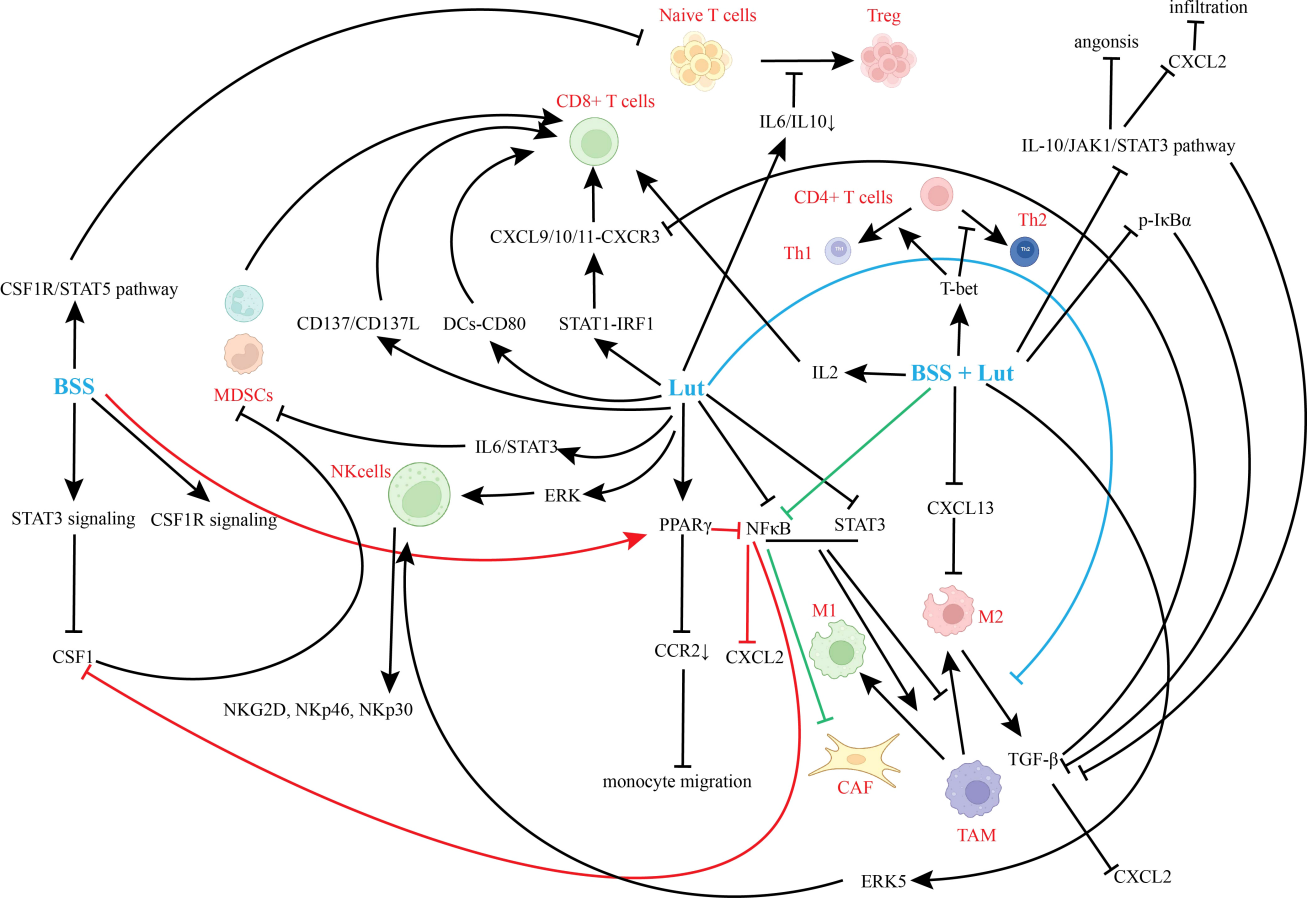
Direct effects on core immune components in the EC TME.

TAMs predominantly exhibit an M2 phenotype in the TME; BSS and Lut reprogram TAMs toward M1 polarization, remodeling the immune landscape. In EC, BSS disrupts signal transducer and activator of transcription 3/colony stimulating factor 1 receptor (STAT3/CSF1R) signaling, reducing colony stimulating factor 1 (CSF1) secretion and inhibiting macrophage colony-stimulating activity (31, 34). Combined BSS/Lut suppress C-X-C motif chemokine ligand 13 (CXCL13) expression, blocking CXCR5 binding—a process correlating with M2 macrophage pro-metastatic effects (9, 31). Lut downregulates C-C motif chemokine receptor 2 (CCR2) via peroxisome proliferator-activated receptor gamma (PPARγ) activation, impairing monocyte migration to the TME. Lut concurrently inhibits STAT3 and NF-κB, abrogating their synergistic M2 polarization promotion (34). In co-culture models, this enhances TAM cytotoxicity and promotes CD8^+^ T cell infiltration (33). BSS activates PPARγ to antagonize NF-κB transcriptional activity, independently inhibiting C-C motif chemokine ligand 2 (CCL2)/CSF1 release through STAT-independent mechanisms (34). Lut reprograms TAMs in breast cancer models (33). Collectively, BSS and Lut suppress chemokines (e.g., CCL2, CSF1) and reverse TAM polarization (M2→M1) by targeting STAT3/6, NF-κB, hypoxia-inducible factor-1α (HIF-1α), and metabolic pathways. Their synergistic blockade of pro-tumorigenic pathways offers novel strategies to overcome TAM-mediated immunosuppression (**Figure 3**).

### 5.2 Regulating the balance of T lymphocyte subsets

BSS and Lut remodel the immune microenvironment by regulating T cell recruitment and activation. In breast cancer, Lut upregulates C-X-C motif chemokine ligand 9/10 (CXCL9/10) transcription via signal transducer and activator of transcription 1-interferon regulatory factor 1 (STAT1-IRF1) activation, enhancing CD8^+^ T cell recruitment to the TME (40, 41). Lut inhibits TGF-β secretion by TAMs, relieving CXCL9/10 transcriptional repression; subsequent CXCL9/10 secretion further recruits C-X-C motif chemokine receptor 3-positive (CXCR3^+^) T cells (40, 41). ascular cell adhesion molecule 1 (VCAM1) inhibits metastatic microenvironment formation (73), while ICAM1-mediated immunological synapses enhance T cell cytotoxicity (34, 39). Lut recruits CD8^+^ T cells via the STAT1-CXCL9/10/11-CXCR3 axis (40, 41). BSS suppresses immunosuppressive microenvironment formation by blocking the TGF-β-peroxisome PPARγ-VCAM1 pathway (34, 73, 76). Combined BSS/Lut exert dual effects: upregulating T cell-recruiting chemokines (CXCL9/10/11) and ICAM1 to promote effector T cell infiltration/activation (34, 40, 41), while downregulating pro-metastatic VCAM1 to inhibit immune evasion (73, 76).

BSS and Lut enhance cytotoxic T lymphocyte (CTL) proliferation, activation, effector functions, and T helper 1 (Th1) responses. Lut promotes CD8^+^ T cell activation/proliferation by augmenting dendritic cell (DC)-CD80 co-stimulation (77). BSS/Lut combinations upregulate IL-2 secretion, driving CTL clonal expansion (77); This promotes CD4^+^ T cell differentiation into Th1 cells via T-box transcription factor (T-bet) upregulation, suppressing T helper 2 (Th2) bias and increasing IFN-γ^+^ CD4^+^ T cells (28, 37, 60). Lut enhances CTL survival/cytotoxicity via CD137/CD137L signaling (40). Collectively, BSS/Lut enhance dendritic cell maturation, activate co-stimulatory axes to promote CTL proliferation/activation/effector functions, and drive Th1 responses.

BSS/Lut inhibit Tregs differentiation/expansion/function and reduce PD-1/Tim-3/LAG-3 expression in exhausted T cells. Lut inhibits naive T cell differentiation into FOXP3^+^ Tregs by downregulating IL-6/IL-10 signaling (35, 37, 39). BSS reduces Treg clonal expansion by interfering with CSF1-R/STAT5 signaling (40, 42). Alleviating Treg-mediated suppression enhances CTL cytotoxic efficiency (39, 40). Coordinated PD-1/Tim-3/LAG-3 downregulation promotes exhausted T cell (T_EX_) conversion to effector T cells (39, 44). BSS inhibits lactate-TGF-β feedback loops to improve acidic microenvironments and block Treg immunosuppression (37, 60), while Lut suppresses enhancer of zeste homolog 2 (EZH2) to hinder natural Treg differentiation into eTregs (42). However, mechanistic evidence derives primarily from endometrial/breast cancer models, warranting further validation of Treg subset regulation (37, 42).

### 5.3 Impact on MDSCs and natural killer cells

Lut inhibits MDSC expansion in the TME by blocking IL-6/STAT3 signaling and suppressing STAT3 phosphorylation. Alleviating MDSC-mediated suppression restores CD8^+^ T cell proliferative capacity (34). BSS reduces MDSC differentiation from bone marrow precursors by downregulating CSF1-R signaling (34).

Lut enhances NK cells cytotoxicity by 30% against tumor cells via ERK signaling activation, upregulating activating receptors (47, 71, 109). BSS/Lut combination therapy inhibits ERK5 activity to relieve transcriptional repression of NK cell activating receptors, upregulating NKG2D, NKp46, and NKp30 expression. This promotes expansion of NKG2D^+^/NKp46^+^ subsets and enhances tumor cell recognition (109). Collectively, BSS/Lut stabilize tumor protein p53 inducible TP53INP2 expression by inhibiting ERK5 activity, upregulate activating receptors (NKG2D/NKp46) and effector molecules (TRAIL/GZMB), enhancing NK cell tumor recognition and cytotoxicity.

### 5.4 Regulation of tumor-promoting CAFs

BSS/Lut reduce tumor invasion by inhibiting CAF activation. BSS/Lut suppress CAF transition from quiescent to activated states by blocking NF-κB signaling (34, 37). They inhibit vasculogenic mimicry formation by suppressing IL-10/JAK1/STAT3 signaling, and downregulate CXCL2 secretion to reduce invasion (9, 37). Additionally, BSS/Lut remodel the immune microenvironment through (1): blocking STAT3 activation by inhibiting IL-10 secretion, downregulating CXCL2-dependent infiltration (9, 34, 35, 37); (2) suppressing TGF-β transcription by blocking nuclear factor of kappa light polypeptide gene enhancer in IκBα phosphorylation (34) (3), downregulating CXCL12 promoter activity, diminishing CAF chemotactic effects on tumor cells (40, 71). Collectively, BSS/Lut downregulate immunosuppressive (TGF-β/CXCL12) and FAP by inhibiting NF-κB, TGF-β/Smad, and Wnt/β-catenin signaling to remodel the TME. This multi-pathway synergy provides novel directions for CAF-targeting combination therapies.

BSS/Lut weaken JAK1/STAT3 signaling by blocking CAF-derived IL-10/TGF-β, inhibiting M2 macrophage polarization (33–35);They downregulate CAF-mediated HIF-1α expression, blocking hypoxia-induced M2 polarization (34). Moreover, BSS/Lut activate LATS1/2-STAT1, promoting MHC-I expression in CAFs to enhance CD8^+^ T cell recognition (47). In EC, BSS/Lut-treated CAFs reduce Treg proportions and lactate secretion, alleviating T cell metabolic suppression (60). In conclusion, BSS/Lut reverse macrophage M2 polarization by blocking CAF-derived immunosuppressive factors, downregulate PD-L1, enhance antigen presentation to restore CD8^+^ T cell cytotoxicity, and improve the metabolic microenvironment to reduce Treg expansion and effector T cell exclusion.

BSS and Lut, as naturally derived bioactive compounds, have shown potential application value in the prevention and treatment of tumors. However, their clinical translation process is hindered by key scientific issues such as extremely low oral bioavailability, unclear pharmacokinetic behavior, and lack of quantitative comparison with standard chemotherapy regimens. Studies have shown that even when high doses of β-sitosterol are administered, the concentration of active drug in systemic circulation remains extremely limited, with an absolute oral bioavailability of only 0.41% (110). In contrast, β-sitosterol nanocomposites have increased oral bioavailability by approximately 3.41-fold (111), but human pharmacokinetic (PK) data on β-sitosterol nanocomposites is severely lacking. Luteolin is similarly constrained by poor physicochemical properties, with inherent limitations such as a short half-life, low water solubility, and low oral bioavailability (112, 113). These characteristics result in failure to reach effective therapeutic concentrations after oral administration and significant fluctuations in blood drug concentration. Compared with BSS, Lut’s nanopreparations provide more quantitative data. Studies have shown that nanoemulsions increased Lut’s oral bioavailability by approximately 2.97-fold (114), while SDS-modified nanocrystals improved Lut’s bioavailability by 3.48-fold (115). Benefiting from excellent membrane permeability, phospholipid complexes have demonstrated the most significant improvement among all nano-strategies, increasing Lut’s relative bioavailability by approximately 5.35-fold (116).

Etoposide combined with cisplatin (EC/EP regimen) is a standard first-line therapy for various solid tumors, with mature and reproducible clinical efficacy data. Compared with BSS and Lut, even with optimal delivery strategies, their systemic exposure may still be far lower than the effective therapeutic concentration. In contrast, the EC regimen ensures 100% bioavailability through intravenous administration (117), which is much higher than the bioavailability of natural products. However, nanoparticle drug delivery systems improve drug efficacy and bioavailability while reducing drug side effects, and surface-modified drug-loaded nanoparticles can precisely target lesion sites (118–120). In addition, nanodelivery systems have shown unique advantages in the treatment of cerebral malaria and inflammation-related diseases (118).

## 6 Immunotherapies targeting regulatory T cells, myeloid-derived suppressor cells, and tumor-associated macrophages

### 6.1 Anti-CD25 antibody-mediated Treg depletion

CD25 is a key surface marker of Treg cells. The use of anti-CD25 monoclonal antibodies to selectively eliminate Treg cells expressing high levels of CD25 is a direct and effective Treg depletion strategy. The Fc segment of the novel anti-CD25 monoclonal antibody RG6292 has been optimized to enhance Treg clearance capacity. In a Phase I clinical study involving multiple advanced solid tumors, RG6292 effectively depleted Treg cells in peripheral blood and tumor tissues both as a monotherapy and in combination with the PD-L1 inhibitor Atezolizumab (121). However, this significant pharmacodynamic effect did not translate into sufficient clinical antitumor efficacy, resulting in its failure to proceed to subsequent clinical exploration. Antibody-drug conjugates (ADCs) represent another developmental direction: by linking potent cytotoxic drugs to CD25-targeting antibodies, precise killing of Tregs can be achieved. Preclinical studies in mouse models have shown that CD25-targeted ADCs effectively deplete Tregs and induce robust antitumor immunity, with relevant drugs (e.g., PF-08046032) having entered Phase I clinical trials for advanced solid tumors (122, 123). Despite the theoretical great potential of anti-CD25 therapy, no studies have investigated anti-CD25 antibodies for EC. Based on its validated Treg clearance capacity in other solid tumors, conducting clinical studies in EC patients—especially those with “hot” immune infiltration but no response to ICIs—is a highly valuable research direction for the future.

### 6.2 Immunomodulatory effects of low-dose cyclophosphamide

Studies have shown that LDC exerts unique immunomodulatory functions rather than traditional cytotoxic effects. Its main mechanism is the selective inhibition or depletion of Treg cells, with minimal impact on effector T cells, thereby improving the TME (124, 125). In addition, relevant studies have been conducted in other tumors such as ovarian cancer and breast cancer (126, 127). However, not all studies have observed the expected Treg depletion effect, suggesting that its role may be influenced by multiple factors such as tumor type, administration regimen, and combination therapy (128). Similar to anti-CD25 antibodies, LDC has not been studied in EC patients. Nevertheless, as an approved and low-cost drug with relatively mature safety profiles and administration regimens, the technical threshold for conducting exploratory clinical studies in EC is low, making it a noteworthy direction for future research.

### 6.3 MDSC inhibitor therapy

All-trans retinoic acid (ATRA), a metabolite of vitamin A, is well-known for its differentiation-inducing therapy in acute promyelocytic leukemia (129–131). In recent years, studies have found that ATRA can also induce the differentiation of MDSCs into mature myeloid cells such as dendritic cells and macrophages, thereby abrogating their immunosuppressive functions (132). In preclinical models, ATRA has been shown to enhance the efficacy of various antitumor therapies. In EC models, ATRA can inhibit MDSC function, thereby significantly potentiating the antitumor effect of anti-PD-L1 immunotherapy, providing a solid theoretical basis for the application of this strategy in gynecological tumors (133). Similarly, despite ATRA’s great potential in targeting MDSCs and significant efficacy in other gynecological tumor models, it has not been studied in EC, which constitutes a clear research gap. Given its well-defined mechanism of action and validated safety in other cancer types, exploring its administration regimen, efficacy, and safety in EC in the future holds important clinical significance.

### 6.4 Application of phosphodiesterase-5 inhibitors

Phosphodiesterase-5 (PDE5) inhibitors represented by Sildenafil and Tadalafil are traditionally used for the treatment of erectile dysfunction, but accumulating evidence indicates they possess significant immunomodulatory activities. Studies have shown that PDE5 inhibitors can effectively suppress the immunosuppressive function of MDSCs by downregulating the expression of ARG1 and nitric oxide synthase 2 (NOS2) in MDSCs (134, 135). Multiple preclinical studies have demonstrated that PDE5 inhibitors can reduce the number and suppressive activity of MDSCs, thereby restoring the cytotoxic functions of T cells and NK cells, enhancing endogenous antitumor immunity, and delaying tumor growth (135–137). The effect of PDE5 inhibitors on MDSC function in EC remains an urgent preclinical research area to be addressed. Verifying the efficacy of PDE5 inhibitors in EC models is a crucial step toward advancing them into clinical trials.

### 6.5 Tumor-associated macrophage repolarization strategy

The CSF-1 and its receptor (CSF1R) signaling axis is a core pathway regulating TAM recruitment, survival, differentiation, and M2 polarization (138). Therefore, CSF1R blockade is a classic strategy for targeting TAMs (139). Studies have found that the expression levels of CSF1 and CSF1R are significantly increased in EC tissues, providing direct pathological evidence for the application of CSF1R inhibitors (140). In preclinical models of various cancers, CSF1R inhibitors (e.g., Pexidartinib, PLX3397) can effectively deplete or repolarize TAMs, reduce M2-type macrophages, and promote CD8+ T cell tumor infiltration, thereby exerting antitumor effects (141, 142). Therefore, verifying the specific efficacy of CSF1R inhibitors in EC models and their regulatory mechanisms on the TME is crucial for guiding their clinical translation.

Despite the clear biological rationale for targeting Tregs, MDSCs, and TAMs, and the advancement of relevant drugs into preclinical or early-phase clinical research stages in multiple solid tumors, specific research data dedicated to EC whether preclinical model validation or clinical trial reports is extremely scarce.

## 7 Safety assessment of BSS/Lut + ICI combinations in EC

ICIs reactivate exhausted T cells and restore the body’s antitumor immunity by blocking inhibitory signaling pathways such as PD-1/PD-L1 or CTLA-4 (143, 144). However, this non-specific immune enhancement disrupts the original immune homeostasis, potentially leading T cells to attack normal tissues of the body, thereby triggering immune-related adverse events (irAEs) (145, 146). irAEs can affect multiple organs including the skin, gastrointestinal tract, liver, lungs, and endocrine system, with severity ranging from mild rashes to life-threatening colitis and myocarditis (147, 148). Combined use of ICIs targeting different molecules (e.g., anti-PD-1 combined with anti-CTLA-4) has been confirmed to significantly increase the incidence and toxicity of severe irAEs (149, 150). This suggests that any drug that may further enhance immune system activity could potentially amplify the risk of irAEs. Currently, studies on β-sitosterol directly regulating T cell activity or affecting the PD-1/PD-L1 pathway are extremely scarce. Search results indicate that existing research mainly focuses on its direct antitumor effects through mechanisms such as inducing apoptosis and regulating cellular signaling pathways (e.g., Akt/GSK-3β) (151). However, one study suggests that liposomal β-sitosterol can inhibit tumor metastasis by eliciting a Th1-type immune response (increasing the production of IL-12, IL-18, and IFN-γ) (152). The Th1-type immune response is the core of ICIs’ antitumor effects, and its overactivation is also closely associated with the occurrence of various irAEs. Therefore, despite the lack of direct evidence, the potential Th1-biased regulatory effect of β-sitosterol provides a theoretical warning for the possible exacerbation of immune responses when combined with ICIs.

Compared with β-sitosterol, the evidence for luteolin’s immunomodulatory effects is more definitive. Preclinical studies have shown that luteolin not only possesses anti-inflammatory and antitumor properties but also directly enhances T cell function (77). A key study found that in a mouse model of hepatocellular carcinoma, luteolin can promote the tumor infiltration of CD8+ T cells and exert a synergistic antitumor effect with PD-1 inhibitors (153). More importantly, luteolin can upregulate key effector cytokines produced by T cells, such as IL-2, TNF-α, and IFN-γ (77). These cytokines are crucial for antitumor immunity, but their uncontrolled elevation—especially that of IFN-γ and TNF-α—is also a key driver in the pathological processes of various irAEs (e.g., colitis, hepatitis). Therefore, luteolin’s characteristic as an “immune accelerator,” when combined with ICIs that “release the brake,” is highly likely to cause the immune system to “lose control,” thereby significantly increasing the risk of irAEs. These cytokines are crucial for antitumor immunity, but their uncontrolled elevation—especially that of IFN-γ and TNF-α—is also a key driver in the pathological processes of various irAEs (e.g., colitis, hepatitis). Therefore, luteolin’s characteristic as an “immune accelerator,” when combined with ICIs that “release the brake,” is highly likely to cause the immune system to “lose control,” thereby significantly increasing the risk of irAEs.

## 8 Challenges and future perspectives

EC-specific studies are scarce, with immunomodulatory research predominantly extrapolated from breast/ovarian cancer models without validation in EC molecular subtypes (POLEmut, microsatellite instability-high [MSI-H], copy number high [CNH]) (40, 154). The estrogen receptor (ERα/PR) network is EC-unique (e.g., IGF1/MYC pathway), yet >80% of studies extrapolate findings from other tumors (99, 154). Immune infiltration (e.g., CD8^+^ TIL density) differs significantly between MSI-H and MMR EC, yet combination therapies lack adequate stratification (9, 155). PD-1 inhibitor response rates are 57% in MSI-H vs 13% in pMMR EC, with unelucidated mechanistic differences (9, 58). Only 30% of studies examine Treg-CTLA4/LAG3 signaling (9), and 80% of EMT-related genes remain unvalidated in EC (156). Associations between dynamic CD8^+^ exhausted T cell markers (HAVCR2/PDCD1) and treatment responses are lacking (9). Systematic pharmacological data are insufficient, with pharmacokinetic (PK) gaps and inefficient targeted delivery. TME-targeted nanocarriers show <15% bioavailability in EC (vs. ~35% in ovarian cancer), with unclear mechanisms (67, 157). High EC stromal stiffness hinders drug penetration, lacking quantitative data (157). Dose optimization criteria are absent combination regimens (e.g., ICIs+ anti-angiogenics) adopt doses from other cancers, increasing grade 3–4 toxicities (hypertension, proteinuria) by 44% (26, 64). Establishing EC organoid-based drug sensitivity platforms (38, 72) and molecular subtype-specific PK/pharmacodynamic (PD) models (58) is urgently needed.

BSS induces M2-to-M1 repolarization, increasing IL-12/IL-10 ratios >2-fold and enhancing phagocytosis (9, 59). Lut inhibits TAMs secretion of IL-6/IL-10 and blocks signal transducer and activator of STAT3 phosphorylation (59, 60). Lut reverses CD8^+^ T cell exhaustion by downregulating T-cell immunoglobulin and TIM-3/PD-1 and restoring IFN-γ secretion (60). Lut reduces FOXP3^+^ Treg proportions, alleviating effector T cell suppression (60, 101). These effects reshape cytokine networks: pro-inflammatory factors (e.g., IL-2, IFN-γ) increase 2-fold (77), while anti-inflammatory factors (e.g., TGF-β/VEGF) decrease 50% (9, 60). In ovarian cancer, BSS/Lut reduce Treg proportions, increase CD8^+^/Treg ratios, and with PD-1 inhibitors achieve 82% tumor suppression rates (59). EC and ovarian cancer share immunosuppressive TME characteristics, supporting mechanistic translatability (9, 46). BSS/Lut upregulate dendritic cell (DC) maturation markers (CD80/CD86), enhancing tumor antigen presentation (59, 101). They induce immunogenic cell death (ICD), releasing HMGB1 and activating DC-T cell axes (77). Lut downregulates PD-L1 on TAMs, enhancing anti-PD-1 antibody binding (59, 101). In MSI-H EC, BSS/Lut-ICI combination therapy increases objective response rates (ORR) to 57% (52, 65). BSS inhibits LPS-induced macrophage hyperactivation, reducing colitis incidence (65, 158). Lut modulates Th17/Treg balance, mitigating autoimmune reactions (159). By remodeling immunosuppressive TMEs, BSS/Lut enhance ICI efficacy and reduce toxicity—particularly for p53abn or MSS EC patients with limited single-agent responses (46, 52, 65).

Research limitations include over-reliance on ovarian/breast cancer models (only 2 orthotopic EC xenograft studies) (9, 22) and lack of molecular subtype stratification (160, 161). Most experiments use subcutaneous xenografts without orthotopic/metastatic models (77, 162). Apply single-cell spatial transcriptomics to dissect BSS/Lut effects on EC-specific T cell clones (65, 163). evaluate 6-month progression-free survival (PFS) of BSS/Lut-pembrolizumab combinations in ICI-resistant patients (52, 164). Baseline TIL density (≥100 cells/mm²) or indoleamine 2,3-dioxygenase 1 (IDO1) circulating tumor DNA clearance could guide stratification (77, 164). Innovations in delivery/precision selection position BSS/Lut regimens as new EC immunotherapy pillars for treatment-refractory patients.

To overcome the lack of targeting specificity of traditional therapies for EC, we propose the following multi-step, refined application roadmap for nanodelivery systems, aiming to achieve precise regulation of EC function. Phase 1: Screening and Validation of Targeting Ligands. The primary task for targeted EC delivery is to identify and utilize molecular markers that are specific to or highly expressed on the EC surface. Future research should first focus on mapping the molecular atlas of EC under different organs (e.g., brain, liver, kidney) and pathological conditions (e.g., atherosclerotic plaques, tumor neovascularization, diabetic retinopathy) using high-throughput technologies such as single-cell RNA sequencing (scRNA-seq) and spatial transcriptomics. Through this approach, we can screen for ideal targets, such as VCAM-1 and ICAM-1 (upregulated in EC under inflammatory conditions) or integrin αvβ3 (specifically expressed on tumor vascular endothelium). Subsequently, it is necessary to develop and validate ligands capable of efficiently binding these targets, such as monoclonal antibodies, aptamers, or small-molecule peptides. Phase 2: Design and Construction of Multifunctional Nanocarriers. Once the target is identified, the next step is to design “smart” responsive nanocarriers. Future carriers should not merely serve as drug containers. For example, pH-sensitive liposomes can be constructed to specifically release anti-inflammatory drugs (e.g., statins or IL-10 gene) in the slightly acidic microenvironment of atherosclerotic plaques. Alternatively, polymeric nanoparticles loaded with siRNA or CRISPR-Cas9 systems can be developed, which, after intravenous injection, accumulate in tumor neovascularization under the guidance of targeting ligands and silence the expression of pro-angiogenic factors (e.g., VEGFR2), thereby achieving precise anti-angiogenic therapy. These carriers can also co-load contrast agents (e.g., quantum dots or superparamagnetic iron oxide) to enable visual tracking of the drug delivery process and efficacy evaluation, forming a “Theranostics” platform. Phase 3: *In Vivo* Validation in Complex Physiological Environments. Ultimately, these advanced nanodelivery systems must be validated in models that highly simulate human physiological and pathological microenvironments. This includes not only traditional animal models but, more importantly, integration with vascularized organoids or “Vessel-on-a-chip” platforms detailed below. In such highly biomimetic *in vitro* models, we can precisely control physical factors such as blood flow shear stress and matrix stiffness, and observe the interaction between nanoparticles and EC, transendothelial transport efficiency, and their impact on downstream cell functions. This step will greatly accelerate the translation from basic research to clinical application, effectively bridging the current “translational gap” between animal experiments and human trials.

### 8.1 Application roadmap of organoid platforms in EC research

The limitations of two-dimensional (2D) culture models have become a major barrier to in-depth understanding of EC function. To address this, we propose a specific roadmap for using organoids and related microphysiological systems as the core platform for future EC research. Phase 1: Construction of Organ-Specific Vascularized Organoids. Future research should shift from monoculture of EC to the construction of organoid models containing functional microvascular networks using induced pluripotent stem cell (iPSC) co-culture technology. For example, in “liver organoids,” iPSCs can be induced to differentiate into hepatocytes, stellate cells, and EC, which self-assemble into a sinusoid-like microvascular network. This model can not only be used to study hepatic drug metabolism and toxicity but also reveal the key role of EC in regulating liver regeneration and fibrosis unprecedentedly. Similarly, the construction of “renal vascularized organoids” will provide a powerful humanized platform for investigating the mechanisms of glomerular endothelial injury in diabetic nephropathy. Phase 2: Integration with Microfluidic Technology for Dynamic “Vessel-on-a-Chip” Upgrading. Static organoids cannot fully simulate the dynamic *in vivo* environment. The core task of the next step is to combine vascularized organoids with microfluidic technology to construct “Organ-on-a-Chip” models. By applying controllable fluid shear stress that mimics physiological or pathological conditions in the chip, we can real-time observe changes in EC morphology, gene expression, and barrier function under hemodynamic stimulation. For instance, high shear stress and turbulent flow at arterial bifurcations can be simulated to study the initiating mechanisms of atherosclerotic plaque formation. Such models will also serve as ideal platforms for testing the interaction between the aforementioned nano-drugs and EC, and evaluating their targeting efficiency under dynamic blood flow. Phase 3: Personalized Disease Modeling and Drug Screening Based on Patient-Derived iPSCs. The ultimate goal of this roadmap is to realize precision medicine. By obtaining somatic cells from specific patients (e.g., those with hereditary hemorrhagic telangiectasia, HHT) and reprogramming them into iPSCs, we can construct personalized “Vessel-on-a-Chip” models carrying the patient’s genetic background. Using this ultimate platform, we can not only delve into the molecular mechanisms by which specific gene mutations lead to EC dysfunction but also perform high-throughput drug screening *in vitro* to “tailor” the most effective treatment plan for the patient, thereby ushering in a new era of personalized therapy for vascular diseases. In summary, EC research is at a critical turning point transitioning from “descriptive” science to “precision regulation and functional remodeling” science. The challenges we face, such as the lack of *in vivo* targeting and insufficient fidelity of *in vitro* models, are precisely the driving forces for technological innovation. We believe that the deep integration of highly specific nanodelivery systems with highly biomimetic humanized organoid/Organ-on-a-Chip platforms will be the core driver leading the paradigm shift in EC research over the next decade. This integration will not only allow us to decipher the mechanisms of EC function and dysfunction at the cellular and molecular levels with unprecedented precision but, more importantly, will construct an efficient and highly personalized translational pathway from basic discovery to clinical application. Ultimately, the ultimate goal of this research paradigm will no longer be merely treating vascular diseases, but achieving active maintenance, repair, and regeneration of tissue and organ functions through precise engineering modification of endothelial function, thereby advancing into a new era of “Precision Vascular Medicine.”

## Glossary

EC: Endometrial Carcinoma
TME: Tumor Microenvironment
TAMs: Tumor-Associated Macrophages
Tregs: Regulatory T Cells
MDSCs: Myeloid-Derived Suppressor Cells
CAFs: Cancer-Associated Fibroblasts
BSS: B-Sitosterol
Lut: Luteolin
ICIs: Immune Checkpoint Inhibitors
TCGA: The Cancer Genome Atlas
POLEmut: Pole Ultramutated Subtype
MSI-H: Microsatellite Instability-High
MMRd: Mismatch Repair Deficient Subtype
NSMP: Non-Specific Molecular Profile Subtype
p53abn: P53 Abnormal Subtype
TILs: Tumor-Infiltrating Lymphocytes
TTK: Tumor Tissue Kinase
MDK: Midkine
NCL: Nucleolin
iNOS: Nitric Oxide Synthase
CTLs: Cytotoxic T Lymphocytes
Arg-1: Arginase-1
NK: Natural Killer Cell
NO: Nitric Oxide
ROS: Reactive Oxygen Species
TCR: T Cell Receptor
IFN-γ: Interferon-Gamma
eTregs: Effector Tregs
dMMR: Mismatch Repair-Deficient
MSS: Microsatellite-Stable
DFS: Disease-Free Survival
CPS: Combined Positive Score
TPS: Tumor Proportion Score
IC: Immune Cell Score
FAP: Fibroblast Activation Protein
ECM: Extracellular Matrix
MMP: Metalloproteinase
myoCAFs: Myofibroblast-Like Cafs
MMP11: Matrix Metalloproteinase-11
TNC: Tenascin C
M-MDSCs: Monocytic Myeloid-Derived Suppressor Cells
NSMP: Non-Specific Molecular Profile
MHC-I: Major Histocompatibility Complex Class I
CXCL12: C-X-C Motif Chemokine Ligand 12
CXCL9: C-X-C Motif Chemokine Ligand 9
CTLA-4: Cytotoxic T-Lymphocyte-Associated Protein 4
DNMT: Dna Methyltransferase
NF-κB: Nuclear Factor Kappa B
PD-L1: Programmed Death-Ligand 1
AP-1: Activator Protein-1
SH2: Src Homology 2
ZEB1: Zinc Finger E-Box Binding Homeobox 1
TCM: Traditional Chinese Medicine
eMSCs: Endometrium-Derived Mesenchymal Stem Cells
PK: Pharmacokinetic
ICD: Immunogenic Cell Death
ORR: Objective Response Rates
